# Impact of *VEGF-C* Gene Polymorphisms and Environmental Factors on Oral Cancer Susceptibility in Taiwan

**DOI:** 10.1371/journal.pone.0060283

**Published:** 2013-04-04

**Authors:** Ming-Hsien Chien, Yu-Fan Liu, Chung-Han Hsin, Chien-Huang Lin, Chun-Han Shih, Shun-Fa Yang, Chao-Wen Cheng, Chiao-Wen Lin

**Affiliations:** 1 Graduate Institute of Clinical Medicine, College of Medicine, Taipei Medical University, Taipei, Taiwan; 2 Wan Fan Hospital, Taipei Medical University, Taipei, Taiwan; 3 Department of Biomedical Sciences, Chung Shan Medical University, Taichung, Taiwan; 4 School of Medicine, Chung Shan Medical University, Taichung, Taiwan; 5 Department of Otolaryngology, Chung Shan Medical University Hospital, Taichung, Taiwan; 6 Graduate Institute of Medical Sciences, College of Medicine, Taipei Medical University, Taipei, Taiwan; 7 Department of Trauma and Emergency Surgery, Wan Fan Hospital, Taipei Medical University, Taipei, Taiwan; 8 Institute of Medicine, Chung Shan Medical University, Taichung, Taiwan; 9 Department of Medical Research, Chung Shan Medical University Hospital, Taichung, Taiwan; 10 Institute of Oral Sciences, Chung Shan Medical University, Taichung, Taiwan; 11 Department of Dentistry, Chung Shan Medical University Hospital, Taichung, Taiwan; China Medical University, Taiwan

## Abstract

**Background:**

Oral cancer, which is the fourth most common male cancer, is associated with environmental carcinogens in Taiwan. Vascular endothelial growth factor (VEGF)-C, an angiogenic/lymphangiogenic factor with high expression levels in tumor tissues, plays important roles in the development of several malignancies. This study was designed to examine associations of five *VEGF-C* gene polymorphisms with the susceptibility to and clinicopathological characteristics of oral squamous cell carcinoma.

**Methodology/Principal Findings:**

Five single-nucleotide polymorphisms (SNPs) of *VEGF-C* were analyzed by a real-time polymerase chain reaction (PCR) in 470 male patients with oral cancer and 426 cancer-free controls. In this study, we found that the *VEGF-C* rs7664413 and rs2046463 polymorphisms were associated with oral-cancer susceptibility but not with any clinicopathological parameters. The *GGACA* or *GACTG* haplotype of five *VEGF-C* SNPs (rs3775194, rs11947611, rs1485766, rs7664413, and rs2046463) combined was also related to the risk of oral cancer. Among 611 male smokers, *VEGF-C* polymorphism carriers who also chewed betel quid were found to have a 14.5–24.2-fold risk of having oral cancer compared to the *VEGF-C* wild-type carrier who did not chew betel quid. Among 461 male betel-quid chewers, *VEGF-C* polymorphism carriers who also smoked had a 2.7–18.1-fold risk of having oral cancer compared to those who carried the wild type but did not smoke.

**Conclusions:**

Our results suggest that the two SNPs of *VEGF-C* (rs7664413 and rs2046463) and either of two haplotypes of five SNPs combined have potential predictive significance in oral carcinogenesis. Gene-environmental interactions among *VEGF-C* polymorphisms, smoking, and betel-quid chewing might alter one's susceptibility to oral cancer.

## Introduction

Oral squamous cell carcinoma (OSCC), a common malignant cancer in the head and neck region, is the fourth most common male cancer and the sixth leading cause of cancer death in Taiwan [1]. The development of OSCC is a multistep process requiring the accumulation of multiple genetic alterations, affected by a patient's genetic predisposition and by environmental influences, including alcohol and tobacco consumption, betel-quid chewing, chronic inflammation, and viral infection [1]–[6]. Expression of a gene may be affected by a single-nucleotide polymorphism (SNP) located within the promoter or other regulatory regions of the gene, and the production or activity of its translated protein is further modulated. SNPs, which are the most common type of DNA sequence variation, occur when a single nucleotide in the shared sequence of a gene differs between members of a species or paired chromosomes in an individual, and are thought to be associated with the development of certain diseases [7]. Genotyping-related SNPs might be a simple and valuable method to predict the risk for and prognosis of cancer. To elucidate the complex process of carcinogenesis and improve the scientific basis for preventive interventions, identifying major genes related to the susceptibility for OSCC should be a priority and effective methods to perform.

Many previous studies demonstrated that tumor-associated angiogenesis and lymphangiogenesis play crucial roles in tumor progression, and angiogenic and lymphangiogenic activities are frequently correlated with tumor growth, regional lymph-node metastasis, distant metastasis, and the prognosis of patients with malignant neoplasms [8]–[10]. The vascular endothelial growth factor (VEGF) family of proteins modulates many endothelial cell functions, especially involving vasculogenesis and angiogenesis [11]. VEGF-A, the first-described member of the VEGF family, induces angiogenesis by activating the related tyrosine kinase receptors, VEGF-R1 and VEGF-R2, on endothelial cells [11], [12]. While VEGF-A plays a paramount role in tumor angiogenesis, VEGF-C was characterized as an essential lymphangiogenic factor that promotes cancer metastasis [13]–[15]. VEGF-C is a ligand for both VEGF-R3 and VEGF-R2, but has a higher affinity for VEGF-R3 [12]. VEGF-R3 is mainly expressed by lymphatic endothelial cells. VEGF-C causes phosphorylation of VEGF-R3, leading to PI3K-dependent Akt activation and protein kinase C (PKC)-dependent activation of the p42/p44 mitogen-activated protein kinase (MAPK) pathway, thus protecting lymphatic endothelial cells from apoptosis and stimulating proliferation and migration *in vitro* [16]. Moreover, it was recently shown that VEGF-R3 may also drive angiogenesis [17], [18]. The angiogenic VEGF-R3 signal is predominantly active in the setting of angiogenic invasion of tissues, such as occurs with tumors. VEGF-R3 potentiates the effects of VEGF-R2 and may sustain angiogenesis, even in the presence of VEGF-R2 inhibitors [18]. Those studies highlighted the significant biological role of the VEGF-C/VEGF-R3 axis in vascular endothelial cells.

Numerous studies demonstrated that VEGF-R3 is also expressed in a variety of human malignancies [19]–[22], and this phenomenon was reported to be a possible predictive factor to determine the clinical approach, because it is correlated with lymph-node metastasis or poor prognosis in patients with prostatic cancer, endometrial carcinoma, OSCC, and non-small cell lung carcinoma [20], [23]–[25]. The function and molecular mechanism of the VEGF-C/VEGF-R3 axis in cancer cells, however, are not well understood. Previous studies demonstrated that tyrosine phosphorylation of VEGF-R3 in cancer cells stimulates cell proliferation in Kaposi’s sarcoma, malignant mesothelioma, leukemia, and gastric cancer [22], [26]–[28]. Others and ourselves showed that activation of VEGF-C/VEGF-R3 signaling in cancer cells enhances cell mobility and invasiveness and contributes to the promotion of cancer-cell metastasis [20], [27], [29]. These findings, taken together, indicate the importance of VEGF-C signaling in tumor progression (growth, invasion, and metastasis) by acting directly on tumor cells.

Impacts of *VEGF-A* polymorphism on human cancer susceptibility are well documented [30]–[33], but the roles of *VEGF-C* gene SNPs and environmental carcinogens in oral cancer susceptibility and clinical features remain poorly investigated. In this research, a case-control study was performed on five SNPs, which are located in the intron or downstream of the *VEGF-C* gene. Some of these SNPs were reported to be correlated with the risk of preeclampsia [34], osteonecrosis of the femoral head [35], or the survival rate with ovarian cancer [36]. In this study, we analyzed associations among *VEGF-C* gene SNPs, environment risk factors, and oral cancer susceptibility. To our knowledge, this is the first study that clearly demonstrates significant associations of *VEGF-C* polymorphisms with oral carcinogenesis.

## Materials and Methods

### Subjects and Specimen Collection

In 2007–2011, we recruited 470 male patients (mean age of 54.0±11.3 years) at Chung Shan Medical University Hospital in Taichung, Changhua Christian Hospital and Show Chwan Memorial Hospital in Changhua, Taiwan as a case group. For the control group, we randomly chose 426 non-cancer individuals (mean age of 50.5±13.9 years) who visited those same hospitals and thus were from the same geographic area. For both cases and controls, we used a questionnaire to obtain exposure information about betel-quid chewing, tobacco use, and alcohol consumption. Medical information of the cases, including TNM clinical staging, the primary tumor size, lymph node involvement, and histologic grade, was obtained from their medical records. Oral-cancer patients were clinically staged at the time of their diagnosis according to the TNM staging system of the American Joint Committee on Cancer (AJCC). Tumor differentiation was examined by a pathologist according to the AJCC classification. This study was approved by the Institutional Review Board of Chung Shan Medical University Hospital and informed written consent to participate in the study was obtained from each individual.

### Selection of VEGF-C Polymorphisms

In dbSNP database, over 60 SNPs has been documented in the intron or downstream of the *VEGF-C* gene region. To obtain adequate power for evaluating the potential association, we investigated rs3775194, rs11947611, rs1485766, rs7664413, and rs2046463, those with minor allele frequencies ≥5%. Furthermore, these SNPs of *VEGF-C* gene were selected in this study since these SNP was found in the cancer patients.

### Genomic DNA Extraction

Genomic DNA was extracted using QIAamp DNA blood mini kits (Qiagen, Valencia, CA, USA) following the manufacturer’s instructions. We dissolved DNA in TE buffer (10 mM Tris at pH 7.8 and 1 mM EDTA) and then quantified it by measuring the optical density at 260 nm. The final preparation was stored at −20°C and used to create templates for the polymerase chain reaction (PCR).

### Real-time PCR

The allelic discrimination of the rs3775194, rs11947611, rs1485766, rs7664413 and rs2046463 polymorphisms of the *VEGF-C* gene was assessed with the ABI StepOne™ Real-Time PCR System (Applied Biosystems, Foster City, CA, USA) and analyzed using SDS v3.0 software (Applied Biosystems), with the TaqMan assay. The FAM-primers used for analysis of rs3775194, rs11947611, rs1485766, rs7664413 and rs2046463 genes polymorphisms were designed as FAM-5′- ATTTAGCACTATTAACTTCAAG; FAM-5′-TTACTTTTGAGAATGTCA; FAM-5′-CTTTTTGATTGCAGTGTTA; FAM-5′-CTTTACTATACTTTACTTGG and FAM-5′- TTTAGCACACGGTTTAGT, respectively. The final volume for each reaction was 5 µL, containing 2.5 µL TaqMan Genotyping Master Mix, 0.125 µL TaqMan probe mix, and 10 ng genomic DNA. The real-time PCR included an initial denaturation step at 95°C for 10 min, followed by 40 cycles of 95°C for 15 s and 60°C for 1 min.

### Statistical Analyses

Differences between groups were considered significant if *p* values were <0.05. Hardy-Weinberg equilibrium (HWE) was assessed using a goodness-of-fit *Χ^2^*-test for biallelic markers. The Mann-Whitney *U*-test and Fisher’s exact test were used to compare differences in demographic characteristic distributions between the healthy control group and oral-cancer patients. The adjusted odds ratios (AORs) and 95% confidence intervals (CIs) of the association of genotype frequencies with risk and clinicopathological characteristics were estimated using multiple logistic regression models after controlling for other covariates. We analyzed all data with Statistical Analytic System (SAS Institute, Cary, NC, USA) software (vers. 9.1, 2005) for Windows.

## Results

The statistical analysis of demographic characteristics is shown in Table 1. There were significant differences in the distributions of betel-quid chewing (*p*<0.001), alcohol consumption (*p*<0.001), and tobacco use (*p*<0.001) between control subjects and male OSCC patients. To diminish the possible interference of environmental factors, AORs with 95% CIs were estimated by multiple logistic regression models after controlling for other covariates in each comparison.

**Table 1:** Distributions of demographic characteristics in 426 controls and 470 male patients with oral cancer.

Variable	Controls (N = 426)	Patients (N = 470)	Odds ratio (95% confidence interval)	*p* value
**Betel nut chewing**				
No	336 (78.9%)	99 (21.1%)	1.00	
Yes	90 (21.1%)	371 (78.9%)	13.991(10.145–19.293)	*p*<0.001*
**Alcohol consumption**				
No	241 (56.6%)	175 (37.2%)	1.00	
Yes	185 (43.4%)	295 (62.8%)	2.196 (1.680–2.870)	*p*<0.001*
**Tobacco use**				
No	224 (52.6%)	61 (13.0%)	1.00	
Yes	202 (47.4%)	409 (87.0%)	7.435 (5.348–10.336)	*p*<0.001*

Mann-Whitney U test or Fisher’s exact test was used between healthy controls and patients with oral cancer. * Statistically significant, *p*<0.05.

**Figure 1:**
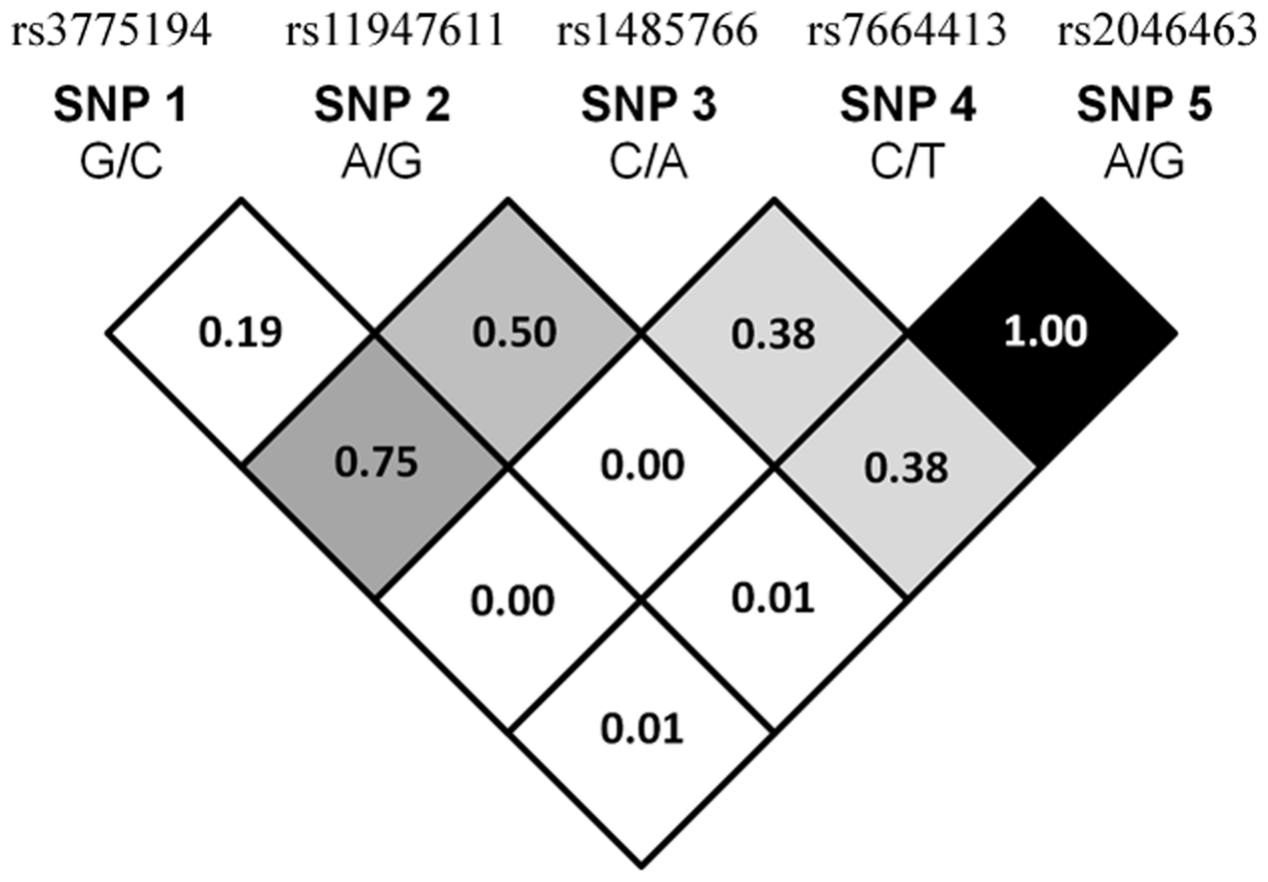
The pairwise linkage disequilibrium (LD) patterns of vascular endothelial growth factor (*VEGF*)*-C* gene. The one observed haploblock, and the pairwise LD measure D’.

In our recruited control group, the frequencies of *VEGF-C* rs3775194 (*p* = 0.844, χ^2^ value: 0.039), rs11947611 (*p* = 0.148, χ^2^ value: 2.090), rs1485766 (*p* = 0.566, χ^2^ value: 0.329), rs7664413 (*p* = 0.115, χ^2^ value: 2.478), and rs2046463 (*p* = 0.115, χ^2^ value: 2.478) were in Hardy-Weinberg equilibrium, respectively. The reconstructed linkage disequilibrium (LD) plot of the five SNPs is shown in Figure 1. We determined one observed haploblock in which rs7664413 and rs2046463 showed 100% linkage disequilibrium in our study. Genotype distributions and associations between oral cancer and *VEGF-C* gene polymorphisms are shown in Table 2. Alleles with the highest distribution frequency for rs3775194, rs11947611, rs1485766, rs7664413, and rs2046463 genes of *VEGF-C* in both of our recruited male oral-cancer patients and healthy control respectively were homozygous for G/G, heterozygous for A/G, heterozygous for C/A, homozygous for C/C, and homozygous for A/A. After adjusting for several variables, there was no significant difference in having oral cancer in individuals with rs3775194, rs11947611, and rs1485766 polymorphisms of the *VEGF-C* gene compared to wild-type (WT) individuals. However, subjects with the *VEGF-C* polymorphic rs7664413 TT genotypes exhibited significantly (*p*<0.05) higher risks of 2.541- (95% CI = 1.071∼6.027), of having OSCC compared to their corresponding WT homozygotes. Moreover, a similar result was also observed in subjects with the *VEGF-C* polymorphic rs2046463 (Table 2).

**Table 2:** Distribution frequency of *VEGF-C* genotypes in 426 healthy controls and 470 male oral cancer patients.

Variable	Controls (N = 426) n (%)	Patients (N = 470)n (%)	Odds ratio (95% confidence interval)	Adjusted odds ratio (95% confidence interval)
**rs3775194**				
GG	302 (70.9%)	355 (75.5%)	1.00	1.00
GC	114 (26.8%)	110 (23.4%)	0.821 (0.606∼1.112)	0.792 (0.515∼1.219)
CC	10 (2.3%)	5 (1.1%)	0.425 (0.144∼1.258)	0.648 (0.159∼2.640)
GC+ CC	124 (29.1%)	115 (24.5%)	0.789 (0.587∼1.061)	0.781 (0.514∼1.188)
**rs11947611**				
AA	180 (42.3%)	185 (39.4%)	1.00	1.00
AG	204 (47.9%)	227 (48.3%)	1.083 (0.819∼1.431)	1.213 (0.817∼1.802)
GG	42 (9.9%)	58 (12.3%)	1.344 (0.859∼2.101)	1.375 (0.714∼2.649)
AG+GG	246 (57.7%)	285 (60.6%)	1.127 (0.863∼1.472)	1.242 (0.853∼1.809)
**rs1485766**				
CC	149 (35.0%)	158 (33.6%)	1.00	1.00
CA	201 (47.2%)	209 (44.5%)	0.981 (0.729∼1.318)	0.873 (0.571∼1.336)
AA	76 (17.8%)	103 (21.9%)	1.278 (0.882∼1.853)	1.153 (0.672∼1.979)
CA+AA	277 (65.0%)	312 (66.4%)	1.062 (0.806∼1.400)	0.946 (0.635∼1.411)
**rs7664413**				
CC	246 (57.7%)	248 (52.8%)	1.00	1.00
CT	163 (38.3%)	181 (38.5%)	1.101 (0.836∼1.451)	1.294 (0.864∼1.939)
TT	17 (4.0%)	41 (8.7%)	2.392 (1.323∼4.325)*	2.541 (1.071∼6.027)*
CT+TT	180 (42.3%)	222 (47.2%)	1.223 (0.939∼1.593)	1.422 (0.967∼2.092)
**rs2046463**				
AA	246 (57.7%)	248 (52.8%)	1.00	1.00
AG	163 (38.3%)	181 (38.5%)	1.101 (0.836∼1.451)	1.294 (0.864∼1.939)
GG	17 (4.0%)	41 (8.7%)	2.392 (1.323∼4.325)*	2.541 (1.071∼6.027)*
AG+GG	180 (42.3%)	222 (47.2%)	1.223 (0.939∼1.593)	1.422 (0.967∼2.092)

Odds ratios and with their 95% confidence intervals were estimated by logistic regression models. Adjusted odds ratios with their 95% confidence intervals were estimated by multiple logistic regression models after controlling for age, betel-nut chewing, tobacco use, and alcohol consumption. * Statistically significant, *p*<0.05.

Interaction effects between environmental risk factors and genetic polymorphisms of *VEGF-C* are shown in Tables 3 and 4. Among 611 smokers, subjects with at least one C allele of rs3775194, one G allele of rs11947611, one A allele of rs1485766, or one T allele of rs7664413, and the betel-nut-chewing habit had respective risks of 14.501-fold (95% CI: 6.899∼30.479), 19.030- (95% CI: 9.239∼39.197), 15.676- (95% CI: 7.413∼33.150), and 24.220- (95% CI: 11.601∼50.566) of having oral cancer. Individuals with either at least one C allele of rs3775194, one G allele of rs11947611, one A allele of rs1485766 or one T allele of rs7664413 or who chewed betel nut had respective risks of 11.688- (95% CI: 6.534∼20.907), 2.827- (95% CI: 1.491∼5.360), 2.670- (95% CI: 1.302∼5.473), and 7.241-fold (95% CI: 3.981∼13.172) of having oral cancer compared to individuals with WT homozygotes who did not chew betel nut (Table 3). Similarly, among 461 betel-quid consumers, subjects with *VEGF-C* polymorphic rs3775194, rs11947611 or rs7664413, genes and who smoked had corresponding risks of 2.695- (95% CI: 1.270∼10.750), 8.066- (95% CI: 2.250∼28.913), and 18.100-fold (95% CI: 5.427∼60.369) of having oral cancer compared to betel-quid chewers with the WT gene who did not smoke (Table 4). In light of the above results, we suggest that *VEGF-C* gene polymorphisms have a strong impact on oral-cancer susceptibility in betel-nut and/or smoking consumers.

**Table 3:** Adjusted odds ratios (AORs) and 95% confidence intervals (CIs) of associations of *VEGF-C* genotypic frequencies and betel-nut chewing among 611 smokers with male oral cancer.

Variable	Controls(*n* = 202) (%)	Patients(*n* = 409) (%)	OR (95% CI)	AOR (95% CI)
**rs3775194**				
a GG genotype without betel-nut chewing	90 (44.6%)	41 (10.1%)	1.00	1.00
b GC or CC genotype or betel-nut chewing	86 (42.6%)	284 (69.4%)	**7.249 (4.663∼11.268)**	**11.688 (6.534∼20.907)**
c GC or CC genotype with betel-nut chewing	26 (12.9%)	84 (20.5%)	**7.092 (3.993∼12.595)**	**14.501 (6.899∼30.479)**
**rs11947611**				
a AA genotype without betel-nut chewing	50 (24.8%)	32 (7.8%)	1.00	1.00
b AG or GG genotype or betel-nut chewing	119 (58.9%)	156 (38.2%)	**2.048 (1.238∼3.390)**	**2.827 (1.491∼5.360)**
c AG or GG genotype with betel-nut chewing	33 (16.3%)	221 (54.0%)	**10.464 (5.888∼18.597)**	**19.030 (9.239∼39.197)**
**rs1485766**				
a CC genotype without betel-nut chewing	44 (21.8%)	28 (6.8%)	1.00	1.00
b CA or AA genotype or betel-nut chewing	109 (54.0%)	140 (34.2%)	**2.018 (1.181∼3.449)**	**2.670 (1.302∼5.473)**
c CA or AA genotype with betel-nut chewing	49 (24.3%)	241 (59.0%)	**7.729 (4.394∼13.594)**	**15.676 (7.413∼33.150)**
**rs7664413**				
a CC genotype without betel-nut chewing	80 (39.6%)	34 (8.3%)	1.00	1.00
b CT or TT genotype or betel-nut chewing	96 (47.5%)	210 (51.3%)	**5.147** **(3.222∼8.221)**	**7.241 (3.981∼13.172)**
c CT or TT genotype with betel-nut chewing	26 (12.9%)	165 (40.4%)	**14.932 (8.392∼26.569)**	**24.220 (11.601∼50.566)**

Odds ratios with their 95% confidence intervals were estimated by logistic regression models.

Adjusted odds ratios with their 95% confidence intervals were estimated by multiple logistic regression models after controlling for age and alcohol consumption.

a Individuals with the wild-type genotype who do not chew betel nut.

b Individuals with either at least one mutated genotype or who chew betel nut.

c Individuals with both at least one mutated genotype and who chew betel nut.

**Table 4:** Adjusted odds ratios (AORs) and 95% confidence intervals (CIs) of associations of *VEGF-C* genotypic frequencies and smoking with male oral cancer among 461 betel-nut consumers.

Variable	Controls (*n* = 90) (%)	Patients (*n* = 371) (%)	OR (95% CI)	AOR (95% CI)
**rs3775194**				
a GG genotype and non-smoker	13 (14.4%)	18 (4.9%)	1.00	1.00
b GC or CC genotype or smoker	52 (56.7%)	272 (72.5%)	**3.809 (1.757∼8.257)**	**4.533 (1.740∼11.808)**
c GC or CC genotype and a smoker	26 (28.9%)	84 (22.6%)	**2.333 (1.009∼5.395)**	**2.695 (1.270∼10.750)**
**rs11947611**				
a AA genotype and non-smoker	9 (10.0%)	11 (3.0%)	1.00	1.00
b AG or GG genotype or smoker	48 (53.3%)	139 (37.5%)	2.369 (0.925∼6.066)	2.897 (0.822∼10.213)
c AG or GG genotype and a smoker	33 (36.7%)	221 (59.6%)	**5.479 (2.111∼14.223)**	**8.066 (2.250∼28.913)**
**rs1485766**				
a CC genotype and non-smoker	6 (6.7%)	11 (3.0%)	1.00	1.00
b CA or AA genotype or smoker	35 (38.9%)	119 (32.1%)	1.855 (0.640∼5.373)	1.406 (0.381∼5.198)
c CA or AA genotype and a smoker	49 (54.4%)	241 (65.0%)	2.683 (0.947∼7.598)	2.940 (0.817∼10.575)
**rs7664413**				
a CC genotype and non-smoker	15 (16.7%)	9 (2.4%)	1.00	1.00
b CT or TT genotype or smoker	49 (54.9%)	197 (53.1%)	**6.701 (2.769∼16.214)**	**10.876 (3.431∼34.471)**
c CT or TT genotype and a smoker	26 (28.9%)	165 (44.5%)	**10.577 (4.198∼26.650)**	**18.100 (5.427∼60.369)**

Odds ratios with their 95% confidence intervals were estimated by logistic regression models.

Adjusted odds ratios with their 95% confidence intervals were estimated by multiple logistic regression models after controlling for age and alcohol consumption.

a Individuals with the wild-type genotype who do not smoke.

b Individuals with either at least one mutated genotype or who smoke.

c Individual with both at least one mutated genotype and who smoke.

We further explored the haplotypes to evaluate the combined effect of the five polymorphisms on oral-cancer susceptibility. The distribution frequencies of *VEGF-C* rs3775194, rs11947611, rs1485766, rs7664413, and rs2046463 haplotypes in our recruited individuals were analyzed. There were five haplotypes with frequencies of >5% among all cases, the most common haplotype in the control was GACCA (35.2%), and it was therefore chosen as a reference. Compared to the reference, two *VEGF-C* haplotypes, GGACA and GACTG, significantly (*p*<0.001) increased the risks for OSCC by 1.568- (95% CI: 1.201∼2.046) and 1.819-fold (95% CI: 1.352∼2.448), respectively (Table 5).

**Table 5:** Distribution frequencies of *VEGF-C* haplotypes in controls and oral-cancer patients.

Variable					Controls(*N* = 852) *n* (%)	Patients(*N* = 940) *n* (%)	Odds ratio (95% confidence interval)	*p* value
rs3775194G/C	rs11947611A/G	rs1485766C/A	rs7664413C/T	rs2046463A/G				
G	A	C	C	A	300 (35.2%)	271 (28.8%)	Reference	
G	G	A	C	A	149 (17.5%)	211 (22.4%)	**1.568 (1.201∼2.046)**	**0.001**
G	A	C	T	G	101 (11.9%)	166 (17.7%)	**1.819 (1.352∼2.448)**	**<0.001**
C	A	A	C	A	86 (10.1%)	73 (7.8%)	0.940 (0.661∼1.337)	0.729
G	A	A	C	A	67 (7.9%)	75 (8.0%)	1.239 (0.858∼1.791)	0.254
G	G	C	C	A	47 (5.5%)	38 (4.0%)	0.895 (0.566∼1.415)	0.635
G	G	C	T	G	35 (4.1%)	37 (3.9%)	1.170 (0.717∼1.911)	0.530
C	G	A	T	G	30 (3.5%)	29 (3.1%)	1.070 (0.626∼1.829)	0.804
G	G	A	T	G	15 (1.8%)	19 (2.0%)	1.402 (0.699∼2.814)	0.342
C	G	C	T	G	11 (1.3%)	7 (0.7%)	0.704 (0.269∼1.843)	0.475
C	A	C	C	A	5 (0.6%)	6 (0.6%)	1.328 (0.401∼4.402)	0.642
G	A	A	T	G	4 (0.5%)	3 (0.3%)	0.830 (0.184∼3.743)	0.809
C	A	A	T	G	1 (0.1%)	3 (0.3%)	3.321 (0.343∼32.117)	0.300
C	G	A	C	A	1 (0.1%)	2 (0.2%)	2.214 (0.200∼24.554)	0.517

To clarify the role of *VEGF-C* gene polymorphisms in the oral-cancer clinicopathologic status, such as TNM clinical staging, primary tumor size, lymph-node involvement, and histologic grade, the distribution frequency of the clinical status and *VEGF-C* genotype frequencies in oral-cancer patients were estimated. No significant association between rs7664413 gene polymorphisms and the clinicopathologic status were observed (Table 6).

**Table 6:** Distribution frequency of clinical status and *VEGF-C* rs7664413 genotype frequencies in 470 patients with oral cancer.

	genotypic frequencies			
Variable	CC (N = 248) n (%)	CT+ TT (N = 222) n (%)	OR (95% CI)	AOR (95% CI)
**Clinical Stage**				
Stage I/II	111 (44.8%)	101 (45.5%)	1.00	1.00
Stage III/IV	137 (55.2%)	121 (54.5%)	0.971 (0.675–1.397)	1.025 (0.672–1.563)
**Tumor size**				
≦ T2	155 (62.5%)	137 (61.7%)	1.00	1.00
> T2	93 (37.5%)	85 (38.3%)	1.034 (0.712–1.502)	1.013 (0.658–1.560)
**Lymph node metastasis**				
No	185 (63.7%)	145 (65.3%)	1.00	1.00
Yes	90 (36.3%)	77 (34.7%)	0.932 (0.638–1.361)	0.883 (0.573–1.359)
**Distant metastasis**				
No	242 (97.6%)	220 (99.1%)	1.00	1.00
Yes	6 (2.4%)	2 (0.9%)	0.367 (0.073–1.836)	0.278 (0.029–2.649)
**Cell differentiation**				
Well	38 (15.3%)	28 (12.6%)	1.00	1.00
Moderately or poorly	210 (84.7%)	194 (87.4%)	1.254 (0.741–2.121)	1.263 (0.700–2.278)

The ORs with 95% CIs were estimated by logistic regression models.

The AORs with 95% CIs were estimated by multiple logistic regression models after controlling for age, betel quid chewing, alcohol consumption, and tobacco use.

> T2: tumor size >2 cm in the greatest dimension.

## Discussion

Alcohol consumption, tobacco smoking, and betel-quid chewing are the main known etiologic factors for oral cancer. In this study, higher ratios were observed of individuals who had chewed betel quid and consumed alcohol and tobacco in the group of OSCC patients (78.9%, 62.8%, and 87.0%, respectively) than control subjects (21.1%, 43.4%, and 47.4%, respectively), which indicates that alcohol and tobacco consumption and betel-quid chewing are highly associated with increased risks of oral cancer. It is well documented that long-term tobacco and betel-quid consumption contributes to oral cancer [3], [4]. Betel-quid constituents might increase protein levels of the c-Fos and c-Jun proto-oncogenes [37]. Tobacco consumption also significantly increases expressions of hypoxia-inducible factor (HIF)-1 [38] and VEGF-C [39] in oral and cervical cancers, respectively. Exposure to environmental carcinogen might partially involve the formation or pathogenesis of oral cancer, but increasing evidence indicates that genomic changes progressively alter cellular phenotypes and might more significantly lead cells to evolve from the preneoplastic stage into cancer [40]. It was reported that the oral mucosa of individuals with the *murine double minute 2 (MDM2)* SNP 309 GG genotype is more susceptible to environmental carcinogen exposure and results in earlier onset of tumor formation [41]. A longer allelic polymorphism of the GT dinucleotide in the *heme oxygenase (HO)-1* promoter and a functional polymorphism in the *nuclear factor kappa B1 (NFKB1)* promoter are both related to the risk of betel-quid-related OSCC [42], [43]. Polymorphisms of several genes were identified as being associated with the risk of oral cancer [44], [45]. It is clear that genetic components may play a pivotal role in carcinogenesis.

VEGF-C is frequently identified in tumor tissues within head and neck squamous cell carcinoma, and the broad expression of the VEGF-C/VEGFR-3 axis in head and neck squamous cell carcinoma suggests involvement in tumor lymphangiogenesis and angiogenesis, promoting tumor growth, and propagation of cancer cells [46]. Data in Table 2 show that individuals with the *VEGF-C* polymorphic rs7664413 TT or rs2046463 GG genotype have higher risks for OSCC compared to the WT genotype. Although the functional importance of these two SNPs has not been tested experimentally, an association with the risk of oral cancer is proposed based on the locations of the analyzed variants. However, in certain genes, an SNP arising in the coding, promoter, or regulatory region may have functional consequences.

The rs7664413 SNP was located on the intron 5 flanking region (−33 nt upstream) of the *VEGF-C* gene. Many alternative splicing cis-regulated elements are located in this region [47]. We further found that the rs7664413 SNP was located in a sequence of a putative exonic splicing silencer (PESS, [TAAGGTATA]). PESSs are cis-regulatory elements that inhibit the use of adjacent splice sites by acting through interactions with members of the heterogeneous nuclear ribonucleoprotein (hnRNP) family and often contribute to alternative splicing (AS). PESSs regulate AS by recruiting factors that directly interfere with the splicing machinery [48]. For example, hnRNP I/PTB binds many exonic splicing silencers and appears to block access to the splicing machinery through protein multimerization [49]. Other evidence supports this observation about two splicing variants (ENST00000280193 and ENST00000507638) reported in the Ensemble database (vers. GRCh37). One encodes the functional VEGF-C protein (NM_005429, 420 amino acids), but the other only processes transcripts (CF128431, without protein production, EST sourced from chondrosarcoma lung metastasis cell lines). Those data suggest that the rs7664413 SNP might affect *VEGF-C* mRNA splicing. However, further specifically designed studies are needed to verify the effects and underlying mechanism of polymorphic rs7664413 on pre-messenger RNA splicing.

The rs2046463 SNP was located downstream of the *VEGF-C* gene but nearby rs7664413 (downstream 5008 nt). As Figure 1 shows, we determined one LD haploblock constituted of rs7664413 and rs2046463, which likely represent dependent genetic signals that affect the risk for OSCC, while other SNPs are outside the haploblock. However, the detailed underlying mechanism needs to be verified by another well-designed experiment.

Interpretations of this study are limited because information on certain oral-cancer risk factors, such as marijuana (cannabis) smoking, medicinal nicotine use, and heredity and familial risks, were not available for the recruited specimens, and this limitation may restrict the adjustment of these possibly confounding factors. In this study, however, the major risk factors for oral cancer, of alcohol and tobacco consumption and betel-quid chewing, were adjusted for in order to estimate the effects of gene polymorphisms on the clinicopathological development of OSCC. In a future study, increasing the specimen number and taking more OSCC risk factors into account in the analysis might precisely validate these findings.

In summary, the *VEGF-C* polymorphic rs7664413 TT or rs2046463 GG genotype might increase the risk for OSCC. The *GGACA* or *GACTG* haplotype of the five *VEGF-C* SNPs (rs3775194, rs11947611, rs1485766, rs7664413, and rs2046463) combined also showed a high risk association with OSCC. Our results suggest that the *VEGF-C* rs7664413 and rs2046463 polymorphic genotypes and haplotype *GGACA* or *GACTG* of the five *VEGF-C* SNPs described above might contribute to predicting the susceptibility to OSCC.
